# Climate anxiety as a call to global justice

**DOI:** 10.3389/fpsyg.2025.1547678

**Published:** 2025-05-12

**Authors:** Batul Hanife, Paolo Cianconi, Francesco Grillo, Alexis Paulinich, Luigi Janiri

**Affiliations:** ^1^Provincial Agency for Health Services, Institute of the Autonomous Province of Trento, Trento, Italy; ^2^Department of Neuroscience, Catholic University of the Sacred Heart, Rome, Lazio, Italy; ^3^Department of History, Anthropology, Religions, Art History, Media and Performing Arts, Sapienza University of Rome, Rome, Italy; ^4^Simple Operating Unit of Health, Clinical and Community Psychology, AUSL Parma, Parma, Italy

**Keywords:** climate change, climate anxiety, eco-anxiety, climate injustice, adaptability

## Abstract

Climate anxiety (or eco-anxiety) is a growing psychological phenomenon linked to the increasing awareness of the environmental crisis caused by climate change. However, it is better understood within the context of the anthropogenic mechanisms that have contributed to pollution and climate change and that are failing to control their consequences, creating a sense of mistrust and uncertainty toward the national and international institutions. Moreover, the impacts of climate change are unequally felt by the rich and the poor also across generations, and policies designed to manage climate change have starkly unequal consequences and the processes by which are decided tend to exclude the poor and the powerless. Nevertheless, even if the groups most at risk for climate change consequences are minorities and marginalized communities, it does not appear that they are the main subjects of criticism and protest, and respondents of color appear to be more likely than white respondents to report feeling traumatized, but less likely to report feeling most of the negative emotions and more likely to feel optimistic and hopeful. Those findings in literature opens the discussion to many questions. Could this apparent discrepancy in climate anxiety reports indicate a difference in historical and cultural perceptions of climate change? Can we consider climate anxiety as a cultural syndrome? Can recognizing these differences in the expression of climate anxiety raise awareness of the unequal impacts of climate change itself and the priority of tackling climate injustice?

Climate anxiety, often referred to as eco-anxiety, is a growing psychological phenomenon linked to the increasing awareness of the environmental crisis caused by climate change (Hickman et al., 2021; Ogunbode et al., 2022; Pearson, 2024). It describes the feelings of dread, helplessness, fear, and concern that individuals experience in response to the ongoing environmental degradation and its potential impacts on the planet. This anxiety can manifest in a wide range of emotional and cognitive responses, from heightened worry about the future to feelings of powerlessness and despair. However, climate anxiety is not purely negative. For many people, the fear and concern about climate change can spur action and various forms of adaptation (Cianconi et al., 2021). It can motivate people to engage in environmental advocacy, adopt sustainable lifestyles, and push for policy change.

Climate anxiety cannot be truly understood without placing it within the context of the anthropogenic mechanisms that have contributed to pollution and climate change and that are failing to control their consequences, creating a sense of mistrust and uncertainty toward the national and international institutions that are supposed to guarantee people's security. All these circumstances ultimately lead to a sense of fear for the future. Which is precisely the main source of anxiety.

At the same time, certain social factors have a marked influence on how climate anxiety manifests itself and on the resources that people have access to when dealing with it, so that the impacts of climate change are unequally felt by the rich and poor also across generations (Dunlap and Brulle, 2015; Pihkala, 2019), knowing that policies designed to manage climate change have starkly unequal consequences and the processes by which are decided tend to exclude the poor and the powerless (Dunlap and Brulle, 2015). In addition many findings across literature suggests climate change is an environmental injustice that is likely to exacerbate existing racialized health inequities such as in the communities of color in the USA which often face disproportionate health risks due to racialized health and socioeconomic disparities unrelated to climate, such as systematic disinvestment in access to quality housing, education, and food (Berberian et al., 2022).

Nevertheless, even if the groups most at risk for climate change consequences are minorities and marginalized communities, it does not appear that these groups are the main subjects of criticism and protest, and the climate sector seems to be unrepresentative in terms of ethnic diversity, such as in the UK (McLoughlin et al., 2024). Moreover, some Authors found in their studies (Sasser and Merchant, 2024) that respondents of color were more likely than white respondents to report feeling traumatized, but less likely to report feeling most of the negative emotions (anger, resentment, depression, powerlessness, anxiety, overwhelm, uncertainty, concern, indifference) and more likely to feel optimistic and hopeful. Especially white women were more likely to report feeling anxious, uncertain, and concerned with respect to climate change in general and the least likely to report any of the positive emotions (optimism, hope, happiness, excitement, determination, and motivation; Sasser and Merchant, 2024).

Some have argued that the prospect of an unlivable future has always shaped the emotional terrain for Black and brown people who have been experiencing existential threats for decades in their history, such as slavery, colonialism, police brutality, and therefore, climate change as the “greatest existential threat of our time” it is not such a unique feeling (Ray, 2021). Similarly, although the most affected populations by climate change are and will be in lower income states, the issue of climate anxiety does not seem to be a topic of interest and discussion while it is reported to be a growing condition within the so-called western world.

Could this apparent discrepancy in climate anxiety reports indicate a difference in historical and cultural perceptions of climate change? Can we consider climate anxiety as a cultural syndrome?

One might speculate that this discrepancy can be attributed to the perceived diminished decision-making authority of minority groups, which consequently leads them to perceive themselves as less efficacious in articulating their grievances or lead them to have a more resigned attitude. Conversely, it could be argued that the priorities they are compelled to address in relation to their disadvantaged status exert a greater influence than other considerations. On the other hand, the apparently lower expression of anxiety about climate change could be attributed to greater resilience due to the cultural and existential challenges already faced.

This leads us to believe that while the construction of structured and repeatable psychotherapeutic interventions to address climate anxiety is undoubtedly beneficial, it is crucial to acknowledge the necessity of personalisation (like in any psychological intervention), which means that a thorough examination of the socio-cultural context in which climate anxiety manifests is of great importance.

Therefore, can recognizing these differences in the expression of climate anxiety raise awareness of the unequal impacts of climate change itself and the priority of tackling climate injustice?

Focusing on climate anxiety solely as a psychological expression of individual distress that needs to be treated according to a standardized model, may downplay the importance of the socio-economic factors involved in its manifestation, and it may also risk depoliticising calls for change toward a more sustainable system (Kałwak and Weihgold, 2022; Verlie, 2024). On the contrary, we cannot talk about climate justice without seriously addressing global injustices and the consequences of neoliberalism.

The apparent discrepancies in the manifestations of climate anxiety among different groups and populations seem to underline these existing differences, which can no longer be ignored.
